# Deferred Radiotherapy After Debulking of Non-functioning Pituitary Macroadenomas: Clinical Outcomes

**DOI:** 10.3389/fonc.2018.00660

**Published:** 2019-01-10

**Authors:** Sarah E. Nicholas, Roberto Salvatori, Alfredo Quinones-Hinojosa, Kristin Redmond, Gary Gallia, Michael Lim, Daniele Rigamonti, Henry Brem, Lawrence Kleinberg

**Affiliations:** ^1^Department of Radiation Oncology and Molecular Radiation Sciences, Johns Hopkins University School of Medicine, Baltimore, MD, United States; ^2^Division of Endocrinology, Diabetes, and Metabolism, Department of Medicine, Johns Hopkins University School of Medicine, Baltimore, MD, United States; ^3^Department of Neurosurgery, Johns Hopkins University School of Medicine, Baltimore, MD, United States

**Keywords:** pituitary, non-functioning, macroadenoma, radiotherapy, outcomes

## Abstract

**Background:** To describe the outcome for a cohort of patients with non-functioning pituitary macroadenomas (NFPMA), managed by debulking surgery with radiation therapy delayed until progression.

**Methods:** Two hundred and sixty-seven patients were treated surgically for pituitary tumors at our institution between 1997 and 2005. One hundred and twenty-six patients met the inclusion criteria of NFPMA. They were followed for at least 2 years.

**Results:** At presentation, 58% of patients had objectively decreased visual function, 66% had endocrine abnormalities, and 46% had headaches. Of the entire cohort, 75% of tumors abutted the optic chiasm and 87% had suprasellar extension. Over a median follow up of 112 months from surgery, 52% of patients had evidence of radiographic tumor progression, and 39% required additional treatment. There was a significant difference freedom from progression and in the number of patients receiving additional treatment with preoperative adenoma size of < 2 vs. ≥2 cm (*p* < 0.05).

**Conclusion:** Close observation with radiation therapy delayed until the time of progression is an appropriate option for patients presenting with initial adenoma size < 2 cm, and can be considered for those with initial sizes up to 4 cm, as the majority of patients do not require further intervention for 10 or more years, thereby meaningfully postponing the risks of radiotherapy.

## Introduction

Pituitary adenomas are a common benign tumor arising from the pituitary gland. They account for 10–15% of all intracranial tumors and are classified by size and hormone secretion (1). Tumors greater than or equal to 10 mm in maximal diameter are considered macroadenomas, and may cause headaches, visual field defects, and hypopituitarism.

Surgery is the first line treatment for symptomatic NFPMA's causing mass effect, but a gross total resection is not always achievable based on the size and surrounding critical structures (2). The role of radiation therapy for NFPMA is controversial. It is sometimes used as an adjuvant to surgery, and other times reserved for treatment of recurrence. A wide range (between 10 and 69%) of patients has been reported to develop progression of NFPMAs within 5–10 years of undergoing surgery (3). Radiation has been shown to reduce the rate of progression, especially when given in the adjuvant setting vs. salvage therapy. For patients receiving radiation therapy, different studies report increased progression free survival at 10 years between 85 and 98% (4–6) and of 92% at 20 years (4). In these studies, prognostic factors include gender, (7) age, and size (5).

Although NFPMA are often treated with radiation after surgical debulking, our practice has included close follow-up, with treatment, that in most cases, was delayed until progression. We present the outcome measured from date of pathologic confirmation for a cohort of NFPMA patients managed with radiotherapy delayed until progression, and we assessed whether consequent to the decision to observe, patients experienced adverse effects of recurrences and subsequent treatment. This information will be of value to patients considering the tradeoffs of the risks of early radiation balanced against the need for later intervention.

## Methods

Between 1997 and 2005, 267 patients with pituitary adenomas were treated surgically. One hundred twenty-six of these patients met the inclusion criteria for non-functioning pituitary macroadenoma (≥10 mm in size) with at least 2 years of follow up at our hospital. Consecutive patients were identified from the pathology department database, which identified patients as having non-secretory adenomas. Repeat surgery or radiation was reserved for recurrence or radiographic growth of the lesion. Data including baseline characteristics and patient demographics was collected retrospectively with Institutional Review Board approval. Only patients receiving 2 years of follow-up at Johns Hopkins Hospital were included as this was a long term follow up study, and many patients come for surgery alone to our institution.

The primary outcome assessed was time to subsequent therapy for the adenoma. Radiographic progression and development of new endocrine and visual field abnormalities, were also retrospectively assessed. Radiographic progression was defined as evidence of radiographic enlargement. New endocrinopathy was defined as the requirement of any new hormonal supplementation in the patient's record. New or increased visual field loss was defined by neuro-ophthalmologic exam. This was compared to baseline status, which was defined as patient status 1 year from date of resection. This was decided to allow for normalization post operatively.

Freedom from progression (FFP) was calculated using the proportion of patients who had reached the defined outcome of new symptoms, including visual symptoms or new endocrinopathy, radiographic progression, or second intervention using GraphPad Prism (version 6). Patients were censored upon reaching an outcome or upon last noted clinic visit. Survival curves were generated by the Kaplan Meier Method. SAS version 9.4 was used for univariate and multivariate analysis.

## Results

Patient characteristics are described in Table 1. The largest proportion of patients had maximum adenoma diameter of 2–2.99 cm. Six patients had 1 surgical resection prior to presenting at our institution; another had 2 surgical resections prior. At baseline, 58% of patients had objective visual field defects, 5.5% of patients had cranial nerve II, III, IV, or VI abnormalities, and 3.1% had optic neuropathy as detected on physical exam. Sixty-six percent had endocrine abnormalities, 46% of patients had headache and 12% presented with pituitary apoplexy. 95.3% of patients underwent trans-sphenoidal resection and 4.7% craniotomy. Post-operative MRI revealed residual tumor in 82% of cases, with a ≥1 cm remnant in 48%. Nine patients received immediate radiation after trans-sphenoidal resection due to patient preference, and were excluded from this study. This left 126 patients to be included in this study.

**Table 1:** Patient characteristics.

**Characteristic**	**Number of patients**	**Percent**
Age (mean)	55 (Range 19–82)	
White	74	59
Black	41	33
Other	11	8
Male	80	63
Female	46	37
Positive smoking history	48	38
No smoking history	68	54
Not reported	10	8
**PRESENTING SYMPTOMS**		
Decreased visual function	73	58
Bitemporal hemianopsia	37	29
Homonymous hemianopsia or quadrantanopsia	29	22
CN (II, III, IV VI) deficit	7	6
Endocrine abnormality	83	65
Headache	58	46
**ADENOMA CHARACTERISTICS**		
Apoplexy	15	12
Size		
1–1.99 cm	22	18
2–2.99 cm	43	34
3–3.99 cm	25	20
>4 cm	9	7
Not otherwise specified (NOS)^*^	27	21
Optic chiasm involvement		
Yes	95	75
No	12	10
Not reported	19	15
Cavernous sinus extension		
Yes	50	40
No	40	32
Not reported	36	28
Suprasellar extension	112	
Yes	110	87
No	2	2
Not reported	14	11

* *Reported as macroadenoma in clinic notes*.

**Figure 1:**
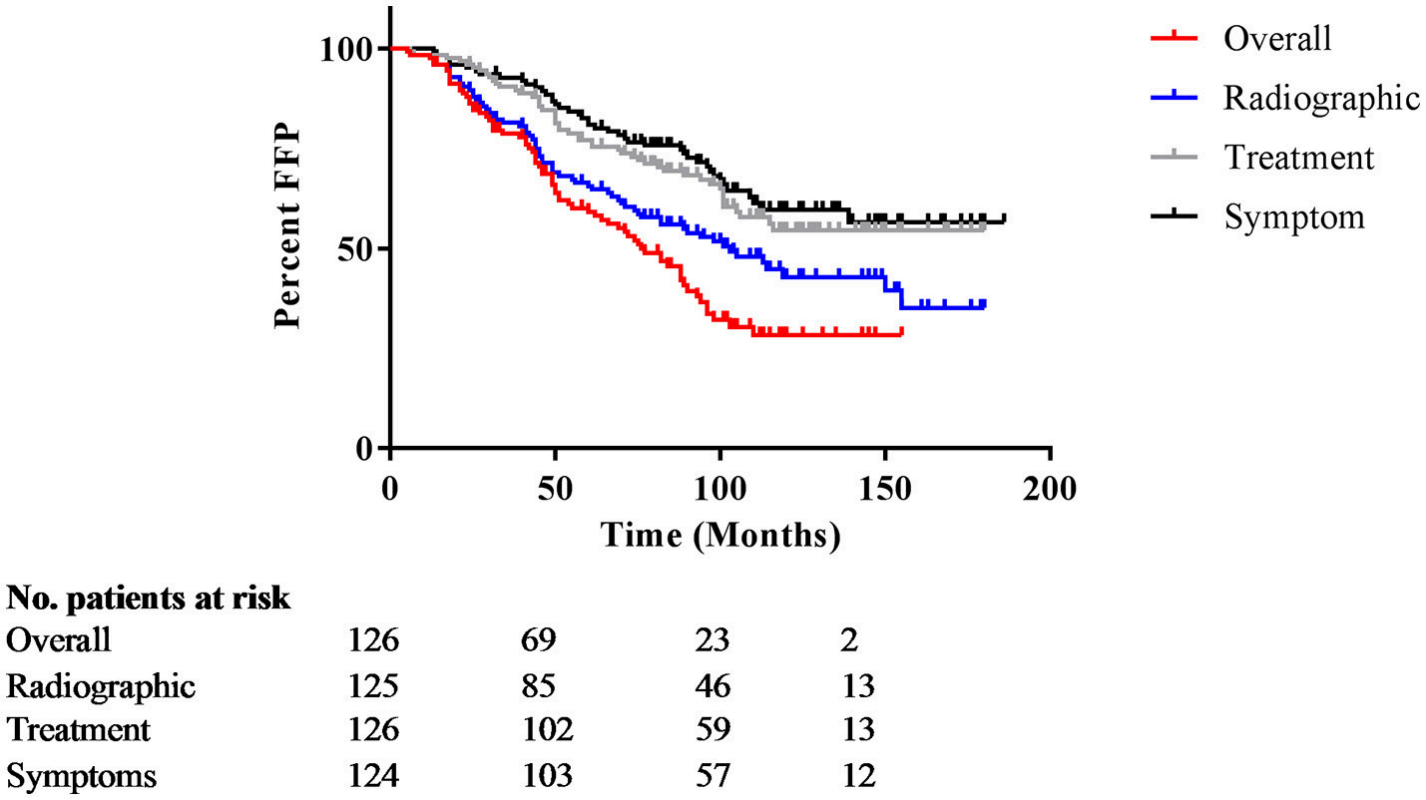
Overall freedom from progression. Kaplan Meier curves for overall freedom from progression, with events defined as new symptoms, radiographic progression, or subsequent intervention. The y-axis represents percentage not progressing; the x-axis is months after initial treatment.

Median follow-up was 112 months (range 24–186 months; Table 2). Twenty percent of patients received a subsequent intervention for the adenoma in the first 5 years after surgery, and 39% required additional treatment during the entire follow-up period. The salvage therapies are described in Table 2. Salvage radiation therapy records were available for 26 patients, and was stereotactic for 4 patients, and standard fractionation for 23 patients. The reason for the first salvage treatment was due to growth in 30 patients, symptoms for 2 patients, a combination of growth and symptoms for 12 patients, and for other reasons for 2 patients. Six patients received adjuvant fractionated radiation within 2–6 months after surgery. A second salvage therapy was indicated for 4 patients with growth, 1 patient with symptoms, 1 surgery occurred for other reasons. Ten patients required a third intervention; 9 of those received radiation and 1 received further surgery. Only 1 patient required a fourth intervention. Overall FFP, time to radiographic progression, and time to intervention are depicted in Figure 1.

**Table 2:** Outcomes for patients including treatment types, symptoms and duration of follow up.

**Factor**	**Number**	**Percent**
Tumor growth	65	52
Salvage treatment	49	39
RT only	19	
Surgery only	20	
Both surgery and RT	10	
New endocrinopathy (*N* = 125)	25	20
New visual field deficits (*N* = 126)	30	24
Median follow up (range)	112 (24–186 months)	

As a result of disease progression or additional therapy, 24% (*n* = 30) of patients developed new or progressive visual field deficits. Twenty-nine of these patients had repeat treatment; for nine, the deficit resolved by last follow up, including one patient who did not received further treatment. Regarding size, 25 patients requiring additional therapy had preoperative size >2 cm, 5 patients with macroadenoma NOS. One patient developed a post-operative stroke resulting in cranial nerve III palsy, another developed post-operative left homonymous hemianopsia. There were no reports of optic neuritis or cranial nerve deficits in the remainder of the patients. Twenty percent of patients developed new endocrinopathies. Of 125 patients with endocrine evaluation at last follow up, 38% had resolution of some or all endocrinopathies. Nine patients in the cohort developed both a new endocrinopathy and worsening visual complaints.

**Figure 2:**
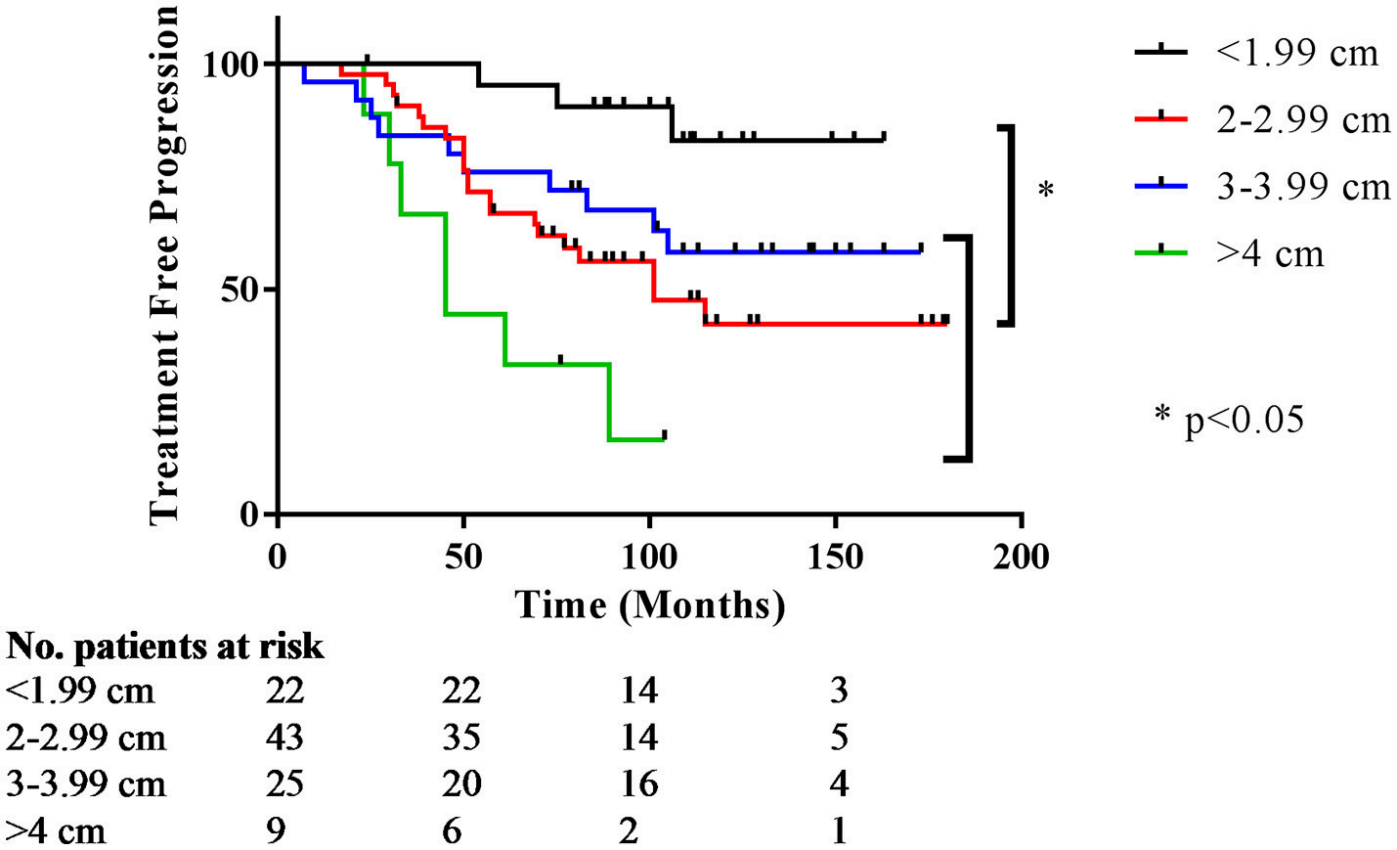
Treatment free progression stratified by preoperative size. Kaplan Meier curves for time to subsequent treatment stratified by preoperative size. The y-axis represents the percentage not requiring treatment; the x-axis is months after initial treatment.

Preoperative size stratification of lesions reveals a significant difference in the number of patients requiring repeat treatment after initial resection. Lesions were classified by maximum dimension, and the survival curves are shown in Figure 2. For the entire cohort, treatment freedom from progression was 80 and 54% at 5 and 10 years, respectively, and overall freedom from progression of 76 months (Table 3). The median salvage treatment free survival for patients with macroadenomas >4 cm prior to surgery was 45 months compared to 115 months for those with sizes from 2 to 3.99 cm (Table 4). In our cohort, pre-operative adenoma size ≥2 cm predicted for a decreased overall freedom from progression (Table 5) and for decreased treatment free progression (Table 6).

**Table 3:** Tumor control.

**Time**	**Overall FFP (*N* = 126)**	**Radiographic FFP (*N* = 125)**	**Treatment FFP (*N* = 126)**
2 year	86%	89%	96%
5 year	59%	65%	80%
10 year	28%	42%	54%
Median	76 months	103 months	undefined

**Table 4:** Freedom from progression based on pre-operative size.

**Size in cm**	**Overall FFP**		**Treatment FFP**		**Median time to treatment**	
	**5 yr**	**10 yr**	**5 yr**	**10 yr**		
1–1.99 (*n* = 22)	84.2	46.4	95	82.9	Undefined	*p* < 0.05
2–2.99 (*n* = 43)	58.5	24.2	66.8	42.3	116 months	
3–3.99 (*n* = 25)	49.1	23.2	76	58		
>4 (*n* = 9)	22.2	0	44.4	16.7		

**Table 5:** Univariate and multivariate analysis overall progression.

	**Descriptive**		**Univariate**		**Multivariate**	
**Variable**	**% With outcome (N)**	***p*-value**	**Hazard ratio**	***p*-value**	**Hazard ratio (CI)**	***p*-value**
Pre-operative size < 2 cm reference	36 (8)	0.0271	2.556 (1.21–5.402)	0.014	2.527 (1.195–5.344)	0.0153
Pre-operative size >2 cm	68 (52)					
Baseline eye problem (Yes vs. No)	71 (34) vs. 49 (38)	0.0148	1.667 (1.042–2.668)	0.033	1.56 (0.928–2.623)	0.0932
Baseline endocrinopathy (Yes vs. No)	50 (12) vs. 59 (60)	0.4319	0.728 (0.391–1.356)	0.3174	0.668 (0.352–1.267)	0.2165

**Table 6:** Univariate and multivariate analysis for salvage treatment.

	**Descriptive**		**Univariate**		**Multivariate**	
**Variable**	**% With outcome (N)**	***p*-value**	**Hazard ratio**	***p*-value**	**Hazard ratio (CI)**	***p*-value**
Pre-operative size < 2 cm reference	14 (3)	0.0027	4.851 (1.495–15.74)	0.0086	2.728 (1.231–6.049)	0.0135
Pre-operative size >2cm	49 (38)					
Baseline eye problem (Yes vs. No)	46 (22) vs. 35 (27)	0.2097	1.273 (0.725–2.236)	0.411	1.747 (1.022–2.987)	0.0412
Baseline endocrinopathy (Yes vs. No)	42 (10) vs. 38 (39)	0.7564	1.031 (0.514–2.068)	0.934	0.721 (0.378–1.375)	0.321

## Discussion

At our institution, we have adopted the approach of treating most NFPMAs with debulking trans-sphenoidal surgery alone, delaying radiotherapy to if and when it would be clinically indicated. With this treatment-free surveillance approach, the long term consequences of recurrence with later need for additional invasive therapy have to be weighed against the side effects of treatment. Previous studies have shown active surveillance to be effective over short term follow up (8). We retrospectively analyzed the outcome of our approach with a longer follow up of almost 10 years to assess long term outcomes.

The majority of our patients observed after debulking did not receive further intervention for five or more years and half did not require further intervention for ten or more years. Although the proportion of patients not having radiographic evidence of growth at 5 years was 65%, somewhat less than the 80% remaining treatment-free, we consider the need for further intervention with surgery or radiation to be the most meaningful metric assessing the success of a strategy primarily designed to postpone the risks and toxicities of further intervention (Tables 2, 3). We found a statistically significant difference in those patients requiring repeat treatment based on pre-operative size. The difference in median time to intervention suggests a way to predict for recurrence based on preoperative adenoma size, and may help patients navigate the treatment options available.

Surgery is the preferred treatment for patients presenting with visual field deficits because it allows for immediate decompression of the optic chiasm. Studies show that surgery alone can improve these symptoms in 84–100% of patients (8, 9). This improvement can be seen as quickly as 2 days post operatively (10). In the initial treatment setting, the risks of delay have been reported in a few studies, each with limited size and with varying conclusions. In a prospective study of 28 patients with NFPMAs, which offered observation at the time of diagnosis, 50% of patients had tumor growth, and 21% of patients underwent surgery due to growth and visual field deficits with resolution of symptoms (9). Another study of 37 patients with incidentally found macroadenomas found that 21 of these patients had tumor enlargement, 10 had visual field changes, and 4 developed apoplexy (11). All but one patient had resolution of visual symptoms with surgery, however, these authors suggest early intervention is justified due to the rate of apoplexy and resulting pan-hypopituitarism (11).

Several papers have discussed the natural history of patients observed after surgical resection, with varying outcomes, and with unclear predictors for growth. In one series of 97 patients observed after surgery, with a mean follow up of 6.3 years, 72% had residual tumor with a 5 and 10-year PFS of 94 and 81% (8). This is a higher PFS rate than in other studies with 5-year local control between 49 and 82% (12, 13). Another recent study of 126 patients by O'Sullivan et al. showed residual tumor in 79% of patients, with a 5-year recurrence free survival of 75.8%; the presence of a postoperative suprasellar remnant was associated with recurrence (14). Our cohort falls within this range, with 5-year progression free survival of 59%, adding to the body of literature of single institution reports.

Visual deterioration is a serious possible (albeit rare) complication of radiation, but severe vision injury including optic neuritis and blindness are reported. Paek et al. reported a series of 68 patients who received surgery and fractionated radiation, ranging from 46 to 52.2 Gy in 1.8–2 Gy fractions, which resulted in 2 cases of radiation induced optic neuritis (15). In a large multicenter study of 512 pituitary adenoma patients, 29 patients were found to have new cranial nerve II deficits following Gamma Knife radiosurgery (GKRS) (16). In our study 24% of patients were noted to have a worsening of visual fields over the follow up period, with one cranial nerve III palsy, and one post-operative left homonymous hemianopsi. Other rare radiation side effects include stroke, which may only be modestly increased by radiation, as well as radiation-induced cancer (17, 18). However, these are lifelong risks that may further increase over a patient's lifetime.

The most common permanent risk of radiation treatment for adenomas identified in the literature remains the development of hypopituitarism. There is a wide range of post radiation hypopituitarism reported in the literature, 0–40% of patients with adenomas developing a new endocrinopathy after radiation therapy (19), and most studies agree that the risk is significant. One study shows up to 50% of patients requiring hormone replacement by 19 years post treatment (3) and more recent series with Gamma Knife radiosurgery show this rate to be as high as 20–28% after a median follow up of 36 and 22 months, respectively (16, 20).

Pomeraniec et al. reported a retrospective matched pair data set of gamma knife eligible patients which provided important outcome information for NFPMA treated with early radiosurgery after surgery, or delayed radiosurgery at the time of documented progression (21). Their data set described that 56% of initially treated vs. 84.4% of those treated later in their disease course had evidence of residual tumor at last follow-up and 5.6 vs. 11.1% (not significant) had evidence of tumor growth (20). Additionally, there was the intriguing finding of an increased risk of endocrinopathy in general, and new endocrinopathy for the group receiving delayed therapy, which was attributed to tumor growth. A multi institutional follow up (22) from the same group showed new endocrinopathy rates of 11.8% in the early GK-SRS group, and 9% in the late GK-SRS group, for a combined rate of 21% over the 6 year follow up. We report a new endocrinopathy rate of 20% with a median follow up of 10 years. Therefore, when comparing the risk of endocrinopathy, watchful waiting in not inferior to adjuvant SRS. The timing of developing an endocrinopathy remains important, which may be of special interest in patients wishing to preserve fertility.

Our study is retrospective and therefor there are several limitations. There were not defined criteria for initiating salvage therapy with radiotherapy or repeat surgery, such that the decision when to intervene after monitoring may be subject to bias. We could not confirm imaging findings as scans were not routinely retained at our institution during the study period, and we therefore could not assess whether the observed changes were definitive or equivocal. Additionally, this retrospective report lacks quantitative and detailed information about new deficits for many patients. The treatment of pituitary adenomas involves coordinated care between surgeons, radiation oncologists, ophthalmologists and endocrinologists. Frequently, patients only followed in some of these departments at our institution, leading to our reliance of qualitative data. Information about biomarkers such as Ki67, which may predict early recurrence, was not available.

Physicians and patients should weigh the risks of new symptoms from tumor and the risks of subsequent resection needed by a proportion of patients at the time of recurrence against those of immediate radiation. It seems to us that in an era with routine availability of both MRI and neuro-ophthalmic follow-up, for an otherwise benign tumor, may be suitably managed with continued years of observation. In our cohort, with a median follow up of 9.3 years, we found that only 39% of patients required salvage treatment, sparing the remainder additional treatment. In addition, in a setting where there is very little long-term data available to define the length of tumor control after radiation, delay of radiation may be beneficial to avoid the time commitment, cost, and rare risk side effects such as neoplasia, and less common, but serious injuries to the brain or brainstem. Adjuvant radiation may be more beneficial for patients to avoid further visual injury, or in cases where the tumor was 3 cm or larger, as these patients had a significantly shorter freedom from progression (Table 5) and this information may provide important guidance for patient decision making. We recommend individualized discussion of delayed treatment after debulking of NFPMA, with the possibility of offering periodical imaging, endocrine, and neuro- ophthalmic follow-up to detect recurrence early in the progression of symptoms. Prospectively collected data would be beneficial in better defining the consequences of progression after observation.

## Data Availability Statement

The raw data supporting the conclusions of this manuscript will be made available by the authors, without undue reservation, to any qualified researcher.

## Ethics Statement

This study was carried out in accordance with the recommendations of the Johns Hopkins School of Medicine Institutional Review Board. The protocol was approved by the Institutional Review Board. Consent was not requires as the research was retrospective in nature, and has no bearing on patient's clinical treatment.

## Author Contributions

SN, RS, and LK contributed to the original concept, acquired and interpreted the data, and contributed to the manuscript draft. AQ-H, KR, GG, ML, DR, and HB contributed to the original concept and critically revised the work. All authors approved the final version to be published.

### Conflict of Interest Statement

The authors declare that the research was conducted in the absence of any commercial or financial relationships that could be construed as a potential conflict of interest.
